# Gap-crossing behavior in a standardized and a nonstandardized jumping stone configuration

**DOI:** 10.1371/journal.pone.0176165

**Published:** 2017-05-03

**Authors:** Karlijn Sporrel, Simone R. Caljouw, Rob Withagen

**Affiliations:** Center for Human Movement Sciences, University of Groningen, University Medical Center Groningen, Groningen, the Netherlands; Univrsidade de Lisboa, PORTUGAL

## Abstract

Over the last years, the omnipresent standardization of playgrounds—the distances between, for example, jumping stones tend to be equal—has been criticized by both scientists and architects. First, it has been argued that standardization fails to do justice to the variability in the children’s action capabilities. Second, it might simplify play in that children repetitively cross over the same distance and, thus, do not have to worry about their movements anymore. In the present study we examined the gap-crossing behavior of children in both a standardized and a nonstandardized jumping stone configuration. Children, between 5 and 10 years of age, were to play in each configuration for two minutes. No significant differences between the configurations were found in the number of gaps the children crossed and the percentage of jumps (ps>0.05). However, more children crossed a gap that they perceived as challenging (i.e. gap width close to their estimated maximum jumping distance) in the nonstandardized configuration than in the standardized one. Interestingly, significant differences were found in variables reflecting the children’s action preparation—the variation in both the time on a jumping stone and the numbers of steps on it was bigger in the nonstandardized playground than in the standardized one (ps<0.05). The implications of these findings are discussed for both the design of playgrounds and the academic discussions about them.

## Introduction

Standardization abounds in playgrounds. Indeed, the distances between the crossbars in a monkey bar or a ladder to a glide tend to be the same. This standardization has been central in the design of playgrounds for many decades. After the Second World War, the Dutch architect Aldo van Eyck developed more than 700 playgrounds in Amsterdam to facilitate the playing behavior of children. Among the elements that figured in his designs were the so-called jumping stones. Although one can arrange these stones in an infinite number of ways, Van Eyck tended to place them in a symmetrical form (i.e. a figure eight) with only two different gap widths for the child to cross [1–3].

Over the last years, this standardization of playgrounds has received critique from both scientists and architects (e.g., [4–6]; see also [7] for an early critique). In a study on how children create their own jumping stone playgrounds, Jongeneel et al. [4] found that the vast majority of children constructed a configuration with varying gap widths. In the discussion of their paper, they argued that such nonstandardized configurations are to be preferred to standardized ones for two reasons. First, following earlier studies on playing behavior [8–10], Jongeneel et al. [4] adopted an affordance perspective. The concept of affordances was introduced by Gibson [11] to refer to the action possibilities in an environment of an animal. For children, for example, a plateau affords jumping upon and a ball affords throwing. Crucially, affordances exist by virtue of the relationship between the properties of the environment and the action capabilities of the actor. For instance, whether a gap is crossable for a child (and how challenging it is for her) depends on the gap width relative to the child’s maximum jumping distance. Following this perspective, Jongeneel et al. [4] argued that a great variety of distances in playground equipment is beneficial because it suits the different action capabilities of children. That is, it affords playing for children who vary in how far they can jump, step, reach, and so on. Also, it allows children to vary in how challenging their playing behavior is—they can alternate between affordances that are easy to utilize and affordances that are more challenging for them. The second reason Jongeneel et al. [4] articulated is that because nonstandardized playgrounds include a variety of distances, they invite children to perform actions in multiple ways. And current thinking in human movement sciences suggests that this is beneficial for their motor development (e.g., [12–15] but see [16]).

Recently, the landscape architect Nebelong [5] formulated a critique on playgrounds that is in keeping with the last point. In her plea for nature playgrounds, she took aim at the omnipresent standardization of playgrounds.

I am convinced that ‘risk-free’, standardized playgrounds are dangerous just in another way from those with obvious risks. When the distance between all the rungs in a climbing net or a ladder is exactly the same, the child has no need to concentrate on where he puts his feet. Standardization is dangerous because play becomes simplified and the child does not have to worry about his movements. (…) The ability to concentrate on estimating distance, height and risk, for example, requires a lot of practice and is necessary for a person to be able to cope successfully with life. (p. 30)

Although several arguments for nonstandardized playgrounds have been forwarded, there is, as far as we know, no study in which the playing behavior in such a playground is compared to that in a standardized playground. In the present study we therefore examined the gap-crossing behavior in two jumping stone configurations: one in which the gaps widths vary substantially, and one in which there are two gap widths. Do children indeed play differently in these configurations? To understand the gap crossing behavior and to determine whether children select gaps that are challenging for them (or that they perceive as challenging), we measured the children’s (perceived) action boundaries for both stepping and jumping. In addition, we also examined the children’s time on the jumping stone and the number of steps on it. These dependent measures provide a window into the action preparation of the children. Hence, if children indeed “worry” more about their movements in the nonstandardized playground, as Nebelong [5] suggested, this is likely to be reflected in these measures.

## Materials and method

### Participants

The study was approved by the ethics committee of the Center for Human Movement Sciences of the University of Groningen and the University Medical Center Groningen. In the fall of 2015, participants were recruited amongst the children of employees of the University of Groningen. Both parents (or guardians) gave written informed consent for their children to participate prior to the study. In total, twenty-five children (fifteen girls, ten boys) between five and ten years of age (7.67 ±1.47) participated in this study. The height of the children was 134.21 cm (±10.73), and their leg length was 68.04 cm (±5.07). One child was excluded from the data analyses because her playing behavior was not completely recorded due to technical problems.

### Design and procedure

**Fig 1:**
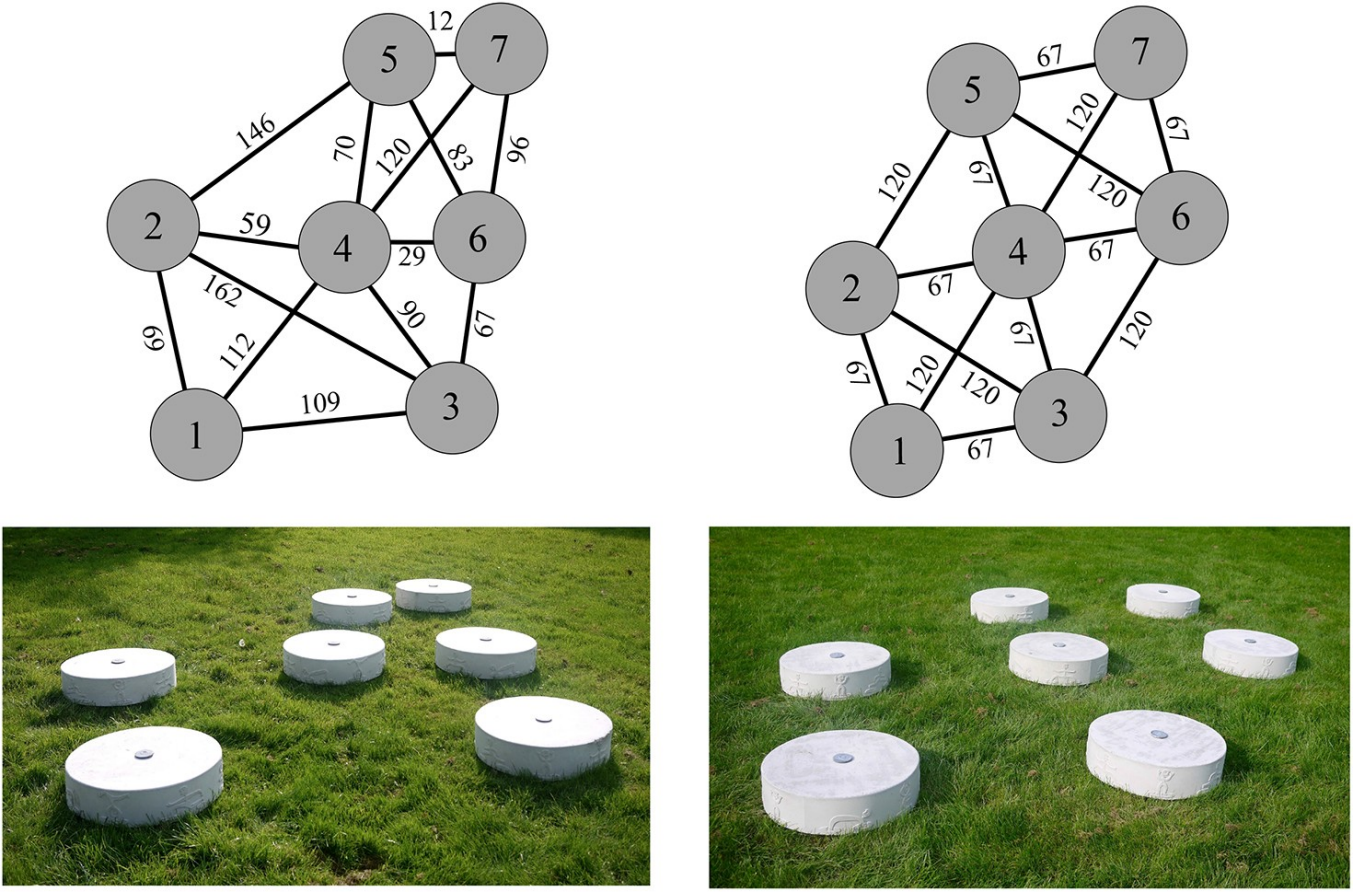
Photo and top view of the standardized (right) and nonstandardized (left) jumping stone configuration with the gap widths in cm.

The study took place on a public grass court on which two jumping stone configurations were placed (see Fig 1). The configurations were surrounded by houses, greenery, and benches, the latter of which were designed by the artist Lambert Kamps. The standardized configuration was based on the design of Aldo van Eyck’s jumping stone playgrounds that was mentioned in the introduction. The nonstandardized configuration and the distances therein were inspired by the configurations that the children had created during the study of Jongeneel et al. [4]. Consequently, this configuration had a great variety of gap widths. Both configurations consisted of seven jumping stones made from concrete with a roughened top surface to prevent slipping. The jumping stones had a diameter of 60 cm and a height of 25 cm. The edges of the stones were rounded off to prevent injuries in case of occasional falls. One camera (GoPro Hero4 Silver) was used to videotape the gap-crossing behavior of the children.

When the children arrived at the playground, they were allowed to play freely for a couple of minutes to get familiarized with the playground. Then each child played individually for two minutes in each of the two configurations, with a short break between the two play phases. Half of the children started in the standardized configuration, the other half started in the nonstandardized configuration. The children were to start on the middle stone and to step or jump from one stone to another without touching the ground. They were free in choosing which gaps they crossed. When a child accidentally fell from a stone, she was encouraged to continue playing on the jumping stones.

To gain insight into the children’s gap-crossing behavior and to determine whether they opted for gaps that are challenging for them to cross (or that they perceived as challenging), we measured for each child the perceived and actual stepping and jumping capabilities after the playing phases. To that end, we used a similar method as Jongeneel et al. [4]. Two round pieces of carpet were used with the same diameter as the stones of the playground (60 cm). One piece of carpet was fixed, the other one could be moved. The children started with estimating how far they could step and jump. After both estimations, we determined their actual stepping and jumping capabilities. For both their estimations and their actual performances, the children started with stepping followed by jumping.

Prior to the stepping and jumping estimations, the experimenter demonstrated that a step means that one of the feet has contact with one of the stones during the whole movement, while in jumping there would be a period in which the child would ‘fly’. The starting distance between the two carpets was set on 75 cm in the stepping condition and on 100 cm in the jumping condition (cf. [4]). After the demonstration, the child was to stand on the fixed piece of carpet, with the toes at the edge of the carpet, and estimated whether she could cross the gap. When she thought she was able to cross the gap, the gap size was increased by 5 cm, whilst the distance was decreased by 5 cm when she thought it was too far. This process continued until the child estimated she could not step or jump any further.

To determine the maximal stepping and jumping distances, the same protocol was followed but now the children had to actually perform the step or jump. The distance between the two pieces of carpet was set on the estimated stepping and jumping distance for that child. If the child successful crossed the gap, the distance between the carpets was increased by 5 cm. If a child did not succeed in crossing the gap within three attempts, the distance was decreased by 5 cm. This process was reiterated until the maximum stepping or jumping distance was reached.

Lastly, the child’s standing height and leg length were determined using Warren’s [17] method. The child had to stand straight against a wall, followed by sitting down with straight legs and their back against the wall. The leg length was computed by subtracting the sitting height from the standing height.

### Analyzing the playing behavior

The videotapes of the playing behavior were analyzed with the Observer XT Version 11.5 (Nodulus Information Technology, Wageningen, Netherlands). For each child we scored which gaps were crossed, how many times the gaps were crossed, how the gaps were crossed (e.g., jumping or stepping), the number of steps the child made on the jumping stone prior to stepping or jumping, and the time the child spent on the stone. The number of steps on a stone was defined as all the steps a child made on the stone after the first foot landed on it. The time on the stone was measured from the moment the child touched the stone while landing till both feet had no contact with the stone. Stepping across a gap means having at least one of the feet on a stone during the execution of the movement, while in jumping across a gap there should be a period in which the child’s feet had no contact with the stones.

To determine the inter-rater reliability, two independent investigators analyzed the videotapes of five children (20,8% of the whole sample). Intra class correlation revealed that the inter-observer agreement was high for time on the stone (r = .997, p < .001) and number of steps on it (r = .989, p < .001). Also a high agreement was found for which gaps were crossed (Cohen’s Kappa = .970, p < .001) and whether the gaps were crossed by means of jumping or stepping (Cohen’s Kappa = .992, p < .001).

## Results

### Children’s estimations of their action capabilities

Because most of the data did not follow a normal distribution, we decided to analyze the complete data set with nonparametric statistics. The estimated and actual stepping and jumping distances of the individual children are listed in Table 1. Wilcoxon signed-rank tests revealed that the actual maximum stepping distance did not differ from the estimated distance (z = -0.354, p = .723, r = -.051). However, the children underestimated their jumping capabilities (z = -3.092, p = .002, r = -.446). This latter finding is consistent with the results of Prieske et al. [6] and Jongeneel et al. ([4] but see [18–19]), and is important for the later analysis of whether children crossed over gaps that are challenging for them. Indeed, if we assume that playing children select gaps based on their *estimated* action capabilities, it implies that we have to use the perceived maximum jumping distance to determine whether children opt for gaps that are challenging for them.

**Table 1:** The perceived and actual action boundaries for stepping and jumping across gaps of each participant.

Participant	Perceived max. stepping distance (cm)	Actual max. stepping distance (cm)	Perceived max. jumping distance (cm)	Actual max. jumping distance (cm)
1	110	115	130	150
2	110	120	120	140
3	85	80	95	100
4	65	70	90	90
5	95	90	110	90
6	100	95	105	105
7	105	110	115	125
8	110	120	130	160
9	90	85	105	105
10	105	90	110	105
11	100	85	115	120
12	110	100	120	115
13	110	120	125	135
14	115	125	130	135
15	100	110	110	130
16	100	115	115	135
17	100	95	110	130
18	110	125	125	145
19	105	105	120	130
20	105	100	110	115
21	105	95	100	95
22	105	105	110	125
23	115	110	120	140
24	115	115	115	150
Average (SD)	102.9 (11.0)	103.3 (15.1)	114.0 (10.5)	123.8 (19.8)

### Gap-crossing behavior

**Fig 2:**
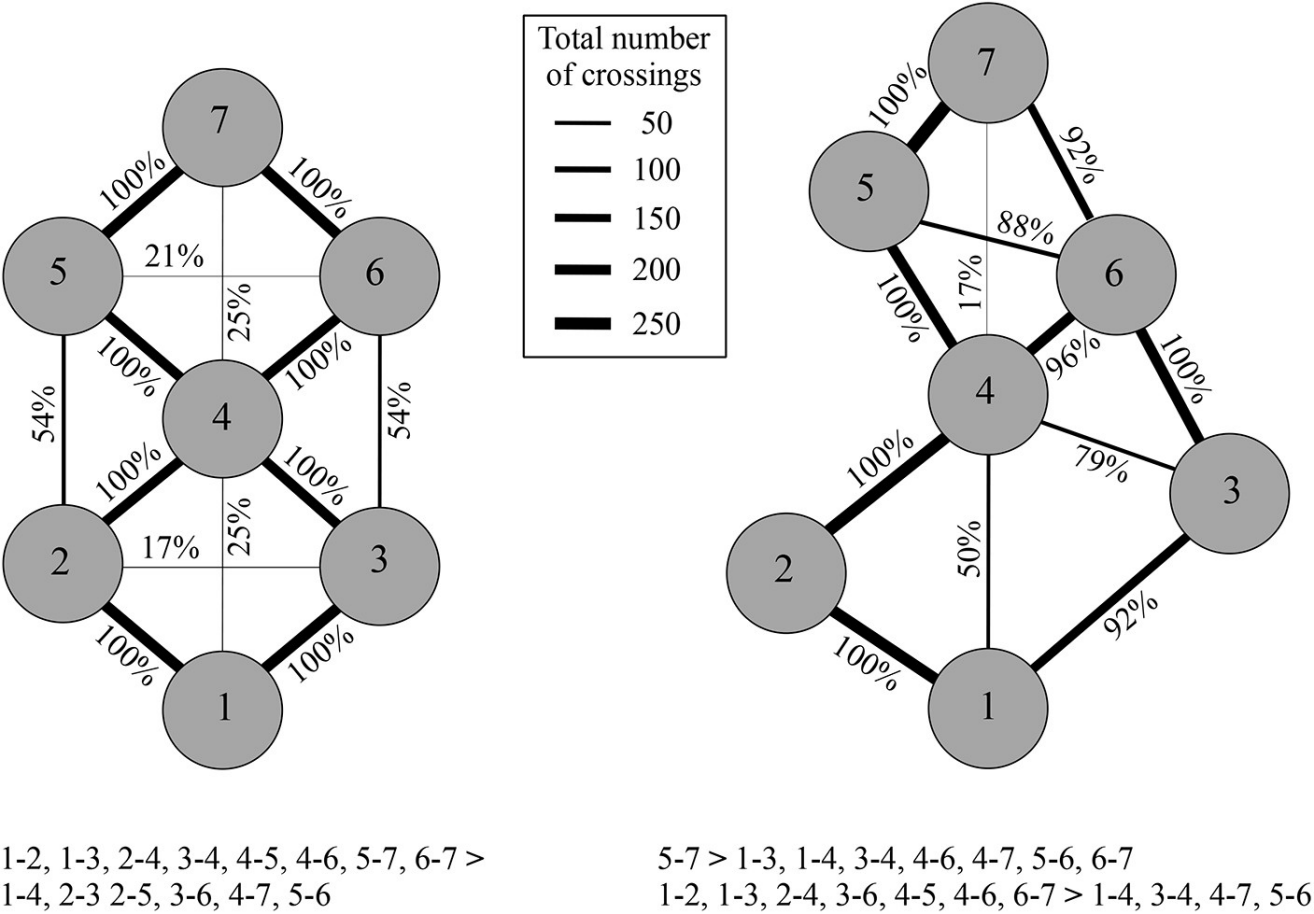
Top view of both the standardized (left) and nonstandardized (right) configuration, with the percentages of children who crossed the gap. The thicker the line, the more frequently the gap was crossed. Significant differences are mentioned below the figure.

We examined for each configuration which gaps were crossed and how many children did so. As one can see in Fig 2 not all children crossed all the gaps. This is to be expected as the participating children varied in their (perceived) action capabilities (see Table 1), and some gaps were too wide to cross for some children or were perceived as such. In the nonstandardized configuration, children crossed over twelve different gaps; in the standardized configuration, on the other hand, children crossed over fourteen different gaps. Interestingly, two wide gaps (i.e., from stone 2 to 3, and from stone 2 to 5) in the nonstandardized playground were never crossed during the study, even though children of the same age and with similar estimated jumping capabilities did build and jump these distances in the study of Jongeneel et al. [4].

To determine whether certain gap widths were crossed more frequently than others, we counted for each child how often she crossed each gap in each of the two configurations. A Friedman test on these frequencies with ensuing post-hoc tests [20] revealed that in the standardized configuration each of the 67 cm gaps was more frequently crossed than each of the 120 cm gaps (Х^2^ (13) = 249.785, p < .001, see also Fig 2). For the nonstandardized configuration, a Friedman test revealed also a differential preference for certain gap widths (Х^2^ (11) = 173.095, p < .001). As one can see in Fig 2, in this nonstandardized configuration the widest gaps are also not among the most frequently crossed. Apparently, in each configuration children crossed the narrowest gaps more frequently than the widest (cf. [6]).

To compare the gap-crossing behavior *between* the two configurations, we determined for each child the percentage of jumps, the number of gaps crossed, and the total distance crossed in each configuration. The medians (and interquartile ranges) of these dependent measures are listed in Table 2. A Wilcoxon signed-rank test revealed that the number of gaps crossed did not significantly differ between the standardized and nonstandardized configurations (z = -0.644, p = .520, r = .092). Also no significant difference was found in the percentage of jumps between the configurations (z = -0.071, p = .943, r = -.010). Taken together, this suggests that the children were equally active in both configurations. However, the children crossed a greater distance in the standardized playground than in the nonstandardized one (z = -2.229, p = .026, r = -.32). This difference can be explained by the fact that the narrow gaps in the nonstandardized configuration were substantially smaller than the narrowest gap in the standardized playground (see Fig 1). And as we have seen earlier, the narrow gaps are crossed more frequently than the widest gaps, giving rise to a significant difference in the total distance crossed between the two configurations.

**Table 2:** The median (and interquartile range) of the number of gaps crossed, the percentage of jumps, the total distance crossed, and the challenge ratio (widest gap crossed/estimated maximum jumping distance) for the standardized and nonstandardized configuration.

	Standardized	Nonstandardized
Number of gaps crossed	76.00 (32.50)	74.00 (30.75)
Percentage of jumps	74.50 (65.00)	65.00 (31.00)
Total distance crossed (m)	53.03 (25.29)	49.23 (24.11)
Challenge ratio	.98 (.35)	.96 (.06)

As touched upon in the introduction, one of the benefits of the nonstandardized configuration is that it affords children with varying jumping capabilities to find a challenging gap to cross. To determine the maximum challenge the child had sought in each of the configurations we divided for each child the maximal gap width that was crossed in each configuration by her estimated maximal jumping distance. A resulting challenge ratio of 0.5, then, shows that the maximum gap width that a child crossed was half the distance she thought she could jump, and a challenge ratio of 1 indicates that the maximum gap width that a child crossed equals her estimated maximum jumping distance. As mentioned earlier, we opted for using the estimated jumping distance (as opposed to the actual) because we found that children underestimated their jumping capabilities, and it is likely that the estimated capability is more important in selecting the gaps than the actual capability (cf. [4]).

The medians of the challenge ratios for the two configurations are listed in Table 2. We found no significant difference in the ratios between the configurations (*z* = -0.401, *p* = .689, *r* = -.058). However, as one can see in Table 2, the interquartile range of the challenge ratio is larger in the standardized configuration than in the nonstandardized one. Inspection of the individual results revealed that in the standardized configuration 66% of the children crossed a gap that was wider than 90% of their estimated jumping distance. Indeed, for some children the 120 cm gaps were too wide to cross. As a consequence the widest gap that they crossed in the standardized configuration was a 67 cm gap, which was relatively easy for them to do so. For these children, however, there were gaps available in the nonstandardized playground that were close to their estimated maximum jumping distance. Interestingly, nearly all children indeed crossed these gaps—in the nonstandardized configuration 92% of the children jumped over a gap that was wider than 90% of their estimated maximum jumping distance. Apparently, children crossed gaps that they perceive as challenging if they are available.

### Action preparation

For each child we computed the median and inter quartile range of both the time spent on a jumping stone and the number of steps on it. We opted for using the median and the inter quartile range because these measures are unaffected by outliers, and the video recordings revealed that occasionally children were simply looking around for some time or playing eeny-meeny-miny-moe. Consequently, children sometimes spent a long time on a stone and made many small steps that arguably do not reflect the preparation of the next action. The medians and interquartile ranges are presented in Table 3.

**Table 3:** The median (and interquartile range) of the individuals’ median and inter quartile range (range) of both the time on the stone and the number of steps on the stone in the standardized and nonstandardized configuration.

	Standardized	Nonstandardized
Median time on stone (s)	1.42 (0.61)	1.42 (0.64)
Range time on stone (s)	0.42 (0.34)	0.59 (0.25)
Median number of steps	2.50 (2.00)	2.00 (1.75)
Range number of steps	1.00 (0.38)	1.75 (1.00)

The Wilcoxon signed-rank test revealed that the time on the stones did not significantly differ between the two configurations (z = 1.484, p = .138, r = .210), and that there was no significant difference in the number of steps on them (z = -0.378, p = .705, r = -.055). Interestingly, the inter quartile ranges of both the time on the stone and the steps on it were significantly larger in the nonstandardized configuration than in the stardardized one (z = -3.349, p = .001, r = -.483, and z = -2.372, p = .018, r = -.342, respectively). This indicates that the action preparation was more variable in the nonstandardized playground than in the standardized playground.

At present it is unclear which factors determine the time on the stone and the number of steps on it. Among the factors that are likely to be involved are the gap that the child is about to cross (relative to her action capabilities), the angle she has to turn, and the gaps that were just crossed. Systematic studies in which these factors are disentangled are needed to settle this issue. However, as a first test we computed Spearman correlations between the children’s estimated maximum jumping distance and the median time on the stone and the median number of steps on it in the nonstandardized playground. We hypothesized that the further a child estimated she could jump, the easier the gaps in the configurations appeared to her, resulting in a reduction in the action preparation (time and number of steps). We indeed found significant negative correlations between the estimated maximum jumping distance and the median time on the stone (r = -.641, p < .001) and the median number of steps on it (r = -.596, p < .001). Similar correlations were found for the standardized configuration (r = -.595, p = .002 for time on the stones; and r = -.658, p < .001 for the number of steps on the stones). Although controlled studies are needed to establish any causal relationships, these correlations suggest that the easier the to-be-crossed gap appears to a child, the less action preparation is needed.

## Discussion

Earlier studies in both architecture [5] and human movement sciences [4,6] have criticized the omnipresent standardization of playgrounds. In the present study we compared children’s gap-crossing behavior in a standardized jumping stone configuration (with two gap widths) and a nonstandardized one (with a variety of gap widths). We found no significant differences between the configurations in terms of the numbers of gaps the children crossed and the percentage of jumps. However, the variation in the time on the jumping stones and the number of steps on them was bigger in the nonstandardized configuration than in the standardized one. In addition, more children crossed a gap that they perceived as challenging in the nonstandardized playground than in the standardized one. In the remainder of this discussion we explore the implications of these results.

### Challenging gap widths

As mentioned in the introduction, one of the benefits of playgrounds with a variety of distances is that they afford playing for children who vary in their action capabilities. Moreover, in such playgrounds individual children can also choose how challenging their actions are—they can jump over gaps that are easy for them to cross but can also go for more challenging ones. In the literature it has been suggested that although children are attracted to challenging affordances, such affordances are not always available in current playgrounds [21–23].

In an earlier study, Prieske et al. [6] examined whether children indeed opt for challenging gaps by letting children play freely in a playscape. Contrary to their expectation, they found that nearly all children crossed the narrower gaps more frequently than the wider ones. The present results are in keeping with this—we also found that in both the standardized and the nonstandardized configuration, the narrowest gaps were among the most frequently crossed. However, the gaps that we created in the jumping stones configurations were wider than those in the playscape of Prieske et al. [6]. In the nonstandardized configuration, the gaps widths were chosen so that they afforded children with varying action capabilities to cross a gap that was close to their (estimated) maximum jumping distance. And interestingly we observed that nearly all children indeed crossed such gaps. Hence, although the narrower gaps were crossed more frequently than the wider ones, children of different ages and with different action capabilities also actualized affordances that they perceived as challenging. This provides an additional argument for the development of nonstandardized playgrounds with a great variety of distances.

### Variability of practice and action preparation

The fact that children crossed over gaps with different widths is supposed to be beneficial for their motor development ([12–15] but see [16]). Indeed, according to the variability-of-practice hypothesis, motor skills mainly develop if they are performed in different ways. As Schmidt [13] put it, “(o)ne of the important thrusts is that children should engage in activities stressing variety in movement patterns (e.g., jump over an object in as many ways as possible)” (p. 275). Obviously, nonstandardized playgrounds with a great variety of distances facilitate this variability of practice more than standardized playgrounds.

In keeping with this principle of human movement sciences, Nebelong [5] recently argued for nonstandardized playgrounds because the varying distances entail that the child has to “worry about his movements” (p. 30). In the present study we assumed that the median time on the jumping stones and the median number of steps on them would increase if the child “worries more” about her movements. However, we found no differences between the configurations in these measures. The variation in both the time on the jumping stones and the number of steps on them, on the other hand, was larger in the nonstandardized configuration than in the standardized configuration. However, the observed negative correlations between the child’s estimated maximum jumping distance and the median time on the jumping stone and the median number of steps on it hint at a simple principle that can explain these differences in variation. Indeed, the correlations suggest that the more challenging the to-be-crossed gap appears to a child, the longer she stays on the jumping stone and the more steps are made on it. Hence, if the time on the stone and the number of steps on it depend on the width of the to-be-crossed gap (relative to the estimated maximum jumping distance), one can expect less variation in these measures in the standardized configuration (with two gap widths) than in the nonstandardized configuration (with a greater variety of gap widths).

Although the observed correlations might look promising, other factors that are likely to be involved need to be scrutinized as well. Indeed, it is well possible that the time on the stone and the steps on it also depend on the angle the child had to turn or on the gap(s) that the child has just crossed. Further studies in which these factors are disentangled are needed to reveal any causal relationship between these factors and the action preparation of the child.

## Supporting information

S1 File DataSporrelCaljouwWithagen. An overview of the individual gap-crossing behavior in both the standardized and the nonstandardized jumping-stone configuration.
(XLSX)
